# Molecular mechanism of interaction between SARS-CoV-2 and host cells and interventional therapy

**DOI:** 10.1038/s41392-021-00653-w

**Published:** 2021-06-11

**Authors:** Qianqian Zhang, Rong Xiang, Shanshan Huo, Yunjiao Zhou, Shibo Jiang, Qiao Wang, Fei Yu

**Affiliations:** 1 Key Laboratory of Medical Molecular Virology (MOE/NHC/CAMS), School of Basic Medical Sciences, Shanghai Institute of Infectious Disease and Biosecurity, Fudan University, Shanghai, China; 2 College of Life Sciences, Hebei Agricultural University, Baoding, China

**Keywords:** Infectious diseases, Infection

## Abstract

The pandemic of coronavirus disease 2019 (COVID-19) caused by severe acute respiratory syndrome coronavirus 2 (SARS-CoV-2) infection has resulted in an unprecedented setback for global economy and health. SARS-CoV-2 has an exceptionally high level of transmissibility and extremely broad tissue tropism. However, the underlying molecular mechanism responsible for sustaining this degree of virulence remains largely unexplored. In this article, we review the current knowledge and crucial information about how SARS-CoV-2 attaches on the surface of host cells through a variety of receptors, such as ACE2, neuropilin-1, AXL, and antibody–FcγR complexes. We further explain how its spike (S) protein undergoes conformational transition from prefusion to postfusion with the help of proteases like furin, TMPRSS2, and cathepsins. We then review the ongoing experimental studies and clinical trials of antibodies, peptides, or small-molecule compounds with anti-SARS-CoV-2 activity, and discuss how these antiviral therapies targeting host–pathogen interaction could potentially suppress viral attachment, reduce the exposure of fusion peptide to curtail membrane fusion and block the formation of six-helix bundle (6-HB) fusion core. Finally, the specter of rapidly emerging SARS-CoV-2 variants deserves a serious review of broad-spectrum drugs or vaccines for long-term prevention and control of COVID-19 in the future.

## Introduction

The pandemic of coronavirus disease 2019 (COVID-19) caused by severe acute respiratory syndrome coronavirus-2 (SARS-CoV-2) infection is still spreading with devasting consequences in mortality and morbidity of human life, as well as the global economy.^1–4^ According to the World Health Organization’s (WHO) newly updated situation report on February 23rd 2021, the COVID-19 pandemic has reached 111,419,939 confirmed cases and claimed 2,470,772 lives, as documented globally in 223 countries worldwide (https://www.who.int/emergencies/diseases/novel-coronavirus-2019). SARS-CoV-2 is transmitted through fomites and droplets during close unprotected contact between the infected and uninfected. Current studies reveal that the most common manifestations of COVID-19 are respiratory symptoms, such as fever, dry cough, and even dyspnea. Severe cases are reported to show sepsis, secondary infections, and organ failure.^5^ More recently, researchers found evidence of gastrointestinal manifestations and potential fecal-oral transmission of COVID-19.^6,7^ The COVID-19 outbreak is the third new acute infectious coronavirus disease to arise in the past two decades, following severe acute respiratory syndrome coronavirus (SARS-CoV) and Middle East respiratory syndrome coronavirus (MERS-CoV),^8–11^ indicating that coronaviruses remain a powerful threat to public health.

**Fig. 1:**
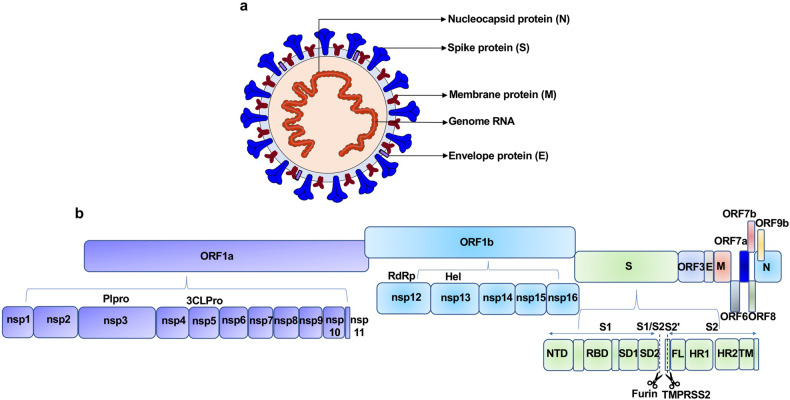
Schematic diagrams of the SARS-CoV-2 virus particle and genome. **a** Four structural proteins of SARS-CoV-2 include Spike protein (S), Membrane protein (M), Nucleocaspid protein (N), and Envelope protein (E). **b** The genome includes ORF1a-ORF1b-S-ORF3-E-M-ORF6-ORF7 (7a and 7b)-ORF8-ORF9b-N in order. Sixteen nonstructural proteins (nsp1–11, 12–16) are encoded by ORF1a and ORF1b, respectively, and six accessory proteins were delineated. Plpro papain like protease, 3CLPro 3C-like proteinase, RdRp RNA-dependent RNA polymerase, Hel Helicase, S encodes NTD N-terminal domain, RBD receptor-binding domain, SD1 subdomain 1, SD2 subdomain 2, FL fusion loop, HR1 heptad repeat 1, HR2 heptad repeat 2, TM transmembrane domain. Dotted line indicates S1/S2 and S2′ site cleavage by Furin and TMPRSS2

SARS-CoV-2 is a single-stranded, positive-sense RNA (+ssRNA) virus, which belongs to lineage B of the genus *Beta-coronavirus* in the *Coronaviridae* family.^12^ The genome size of SARS-CoV-2, which was sequenced recently, is ~29.9 kb, sharing ~78% sequence homology with SARS-CoV.^12,13^ The SARS-CoV-2 genomic RNA includes two major open reading frames (ORFs), ORF1a and ORF1b, encompassing two-thirds of the genome and translated to pp1a and pp1b proteins. The virus genome encodes 2 cysteine proteases, a papain-like protease (PLpro), or nsp3, and a 3C-like protease (3CLpro), or nsp5. These proteases cleave pp1a and pp1b polypeptides into 16 nonstructural proteins.^14,15^ The core of RNA-dependent RNA polymerase (RdRp) consists of nsp12, which is a critical composition of coronavirus replication/transcription. nsp7 and nsp8 significantly increased the combination of nsp12 and template-primer RNA.^16,17^ Notably, the RdRp is one of the most promising drug targets identified to date.^18^ The remaining one-third of the genome has overlapping ORFs, encoding four major structural proteins, including S (spike glycoprotein), N (nucleocapsid protein), M (membrane protein) and E (envelope protein), and some accessory proteins.^15,18^ The S protein consists of the signal peptide (SP), receptor-binding domain (RBD), subdomain 1 (SD1) and subdomain 2 (SD2) in S1 subunit and fusion peptide (FP), heptad repeat 1 (HR1), heptad repeat 2 (HR2), and transmembrane (TM) in membrane-fusion subunit (S2).^19^ The E protein, along with M and N, is known to facilitate virus-like particle formation.^20^ SARS-CoV-2 also encodes accessory proteins, including ORF3, ORF6, ORF7a, ORF7b, ORF8, and ORF9b, which are all distributed among the structural genes (Fig. 1).^14^

SARS-CoV-2 enters into the host cell by direct fusion of the viral envelope with the host cell membrane, or membrane fusion within endosome after endocytosis. Viral entry is initiated by binding RBD of the S protein to the human host cell receptors at the cell surface.^21–25^ One major receptor for SARS-CoV-2 is angiotensin-converting enzyme 2 (ACE2), which is widely expressed in the cells of the lung, intestine, liver, heart, vascular endothelium, testis, and kidney.^26^ Recently, other host receptors and/or co-receptors that promote the entry of SARS-CoV-2 into cells of the respiratory system have been reported. After RBD-receptor interaction, the S protein undergoes proteolytic cleavage, which is then catalyzed by several host proteases, such as furin, TMPRSS2, and cathepsin B/L. Proteolytic processing activates S protein and allows for viral-host membrane fusion, followed by the release of viral RNA into the host cytoplasm. In the cytoplasm, viral RNA utilizes the host and its own machinery to replicate its genetic material and assemble new viral particles.^27,28^ Apparently, SARS-CoV-2 has extremely broad cell tropism. In addition to type II alveolar epithelial cells and ciliated cells in the lungs, SARS-CoV-2 can also infect intestinal epithelial cells and brain cells, leading to intestinal symptoms and brain inflammation.^29–31^

In the setting of the current COVID-19 pandemic, extreme steps have been taken to develop effective prophylactic and therapeutic strategies.^34^ Various efforts are being made globally to shorten the research time of convalescent plasma, vaccines, neutralizing antibodies and other antiviral drugs. In particular, convalescent plasma has been reported as a potential therapy for COVID-19.^32^ Hospitalized COVID-19 patients transfused with convalescent plasma exhibited a lower mortality rate compared to patients receiving standard treatments.^33,34^ The development of SARS-CoV-2 vaccines also has accelerated worldwide.^33–35^ Fortunately, several mRNA and inactivated vaccines have reported clinical protection efficacy and are currently obtained through emergency authorization for vaccination globally to stop the spread of COVID-19^35–38^. Neutralizing antibodies may serve as a potential treatment approach against COVID-19 owing to their excellent neutralizing efficiency and production scale-up. Many studies have reported that multiple antibodies targeting RBD have been screened and shown to have potent neutralizing activity in vitro and in animal models.^43–47^ To date, many neutralizing antibodies have been evaluated clinically.^39,40^ Several drugs, such as hydroxychloroquine, arbidol, and remdesivir, are currently undergoing clinical studies to test their safety and efficacy.^41,42^ In addition, peptides derived from the HR1 and HR2 of S protein have been revealed to possess antiviral activity.^43,44^ Traditional Chinese medicines have also played an important role in curbing this epidemic.^45^ The early combined use of Lianhua Qingwen and antiviral drugs may accelerate recovery and improve the prognosis of patients with moderate COVID-19.^42,46^ Here, we systematically elaborated the interaction between SARS-CoV-2 and host cell factors on the cell surface during membrane fusion. Moreover, we have summarized in detail the current antiviral therapies against SARS-CoV-2 entry into host cells, including antibodies, convalescent plasma, as well as peptide- and small-molecule compound-based antiviral therapies.

## Interaction between SARS-CoV-2 and host receptors

Uncovering the molecular mechanism that underlies the entry of SARS-CoV-2 is one of the most important puzzles in understanding how to block its infection. Coronaviruses enter host cells in three ways: receptor-mediated plasma membrane fusion, receptor-mediated endocytosis, or antibody-dependent viral entry. Receptor proteins on the surface of host cells are crucial for virus attachment on host cells for both fusion and endocytosis. Research on the interactions between SARS-CoV-2 and its receptors has provided novel insight into virus transmissions and has resulted in a solid foundation for the development of novel strategies for clinical prevention and treatment.^47^

### Host receptor ACE2 for SARS-CoV-2 attachment

The cell receptor for SARS-CoV-2 virus is identified as the angiotensin-converting enzyme II (ACE2).^2,48^ As a type I integral membrane protein composed of 805 amino acids,^49^ human ACE2 migrates to the surface of cells after transcription with its N-terminal signal peptide, and it successfully anchors via a C-terminal transmembrane domain.^50,51^ The ACE2 protein contains an N-terminal peptidase domain comprised of two lobes, and when coronaviruses contact the surface of host cells, the spike RBD binds the tips of one lobe to initiate viral entry.^18,22,23,52^

The complex structure of SARS-CoV-2 RBD and ACE2 resolved to less than 3 Å provided detailed and important insights into the molecular basis of virus-receptor interaction.^18,20,23,52^ With two structural domains, a five-stranded, antiparallel β-sheet core subdomain and an external subdomain with an extended flexible loop stabilized by a disulfide bond^53^, SARS-CoV-2 RBD utilizes its external subdomain to recognize the N-terminal peptidase domain of ACE2, similar to that reported for SARS-CoV.^25^ This external subdomain of RBD was named as the receptor-binding motif (RBM), and its two-stranded β-sheet component, also called β-hairpin, forms a concave that cradles the convexity on the surface of ACE2 peptidase domain.

Atomic-level analysis shows the details of protein binding interfaces between SARS-CoV-2 RBD and ACE2 and the amino acid residues in close proximity on both proteins.^20,23,52^ Each ACE2 peptidase domain accommodates a single RBD, with an extended loop region of the RBD spanning the bridge-shaped *α*1 helix of ACE2 peptidase domain. There are three clusters of contacts between these two molecules.^23^ At the N terminus of this bridge-shaped structure, Q498, T500, and N501 residues of the RBD protein form a hydrogen-bond network with Y41, Q42, K353, and R357 amino acid residues of the ACE2 peptidase domain.^23^ At the C terminus of bridge-shaped *α*1 helix, RBD Q474 forms a hydrogen bond with the ACE2 Q24, with RBD F486 interacting with the ACE2 M82 through van der Waals forces.^23^ Moreover, K417 and Y453 on the RBD protein interact, respectively, with D30 and H34 of ACE2 protein.^23^

The broad interface between SARS-CoV-2 RBD and ACE2 is consistent with their extraordinary binding affinity, as determined by Bio-Layer Interferometry analysis or Surface Plasmon Resonance measurement.^18,22,23,52^ Moreover, despite the high degree of structural similarity between SARS-CoV and SARS-CoV-2, SARS-CoV-2 RBD displays more amino acid residues on the binding interface and more contacts by the residues, resulting in a four-fold stronger binding affinity compared with that of SARS-CoV RBD.^52^ The calculated Kd values for SARS-CoV-2 and human ACE2 binding affinity by different groups are all in the nanomolar level, ranging from 5 nM to 95 nM^47^ This outstanding binding affinity between SARS-CoV-2 RBD and its receptor ACE2 may explain the high infectivity and global spread of COVID-19.

Structural analysis by both X-ray and cryo-electron microscopy (cryo-EM) indicates that one RBD molecule binds to one ACE2 receptor protein molecule.^18,20,22,52,54–56^ However, reality is more complex. SARS-CoV-2 S glycoprotein is revealed to adopt a homotrimeric conformation with an RBD subunit presented on each monomeric unit. The RBD can exhibit two distinct conformational states, either “up” or “down” conformation.^48,55,57,58^ When the RBD points downward, its interaction with ACE2 is inhibited by steric hindrance. The position switching from “down” to “up” conformation exposes the receptor binding site, thus facilitating ACE2 interaction.^58–60^ More than 16 amino acid residues in the opened RBD interact with human ACE2, while, conversely, 21 amino acid residues in human ACE2 receptor interact with SARS-CoV-2 RBD.^20,52^ On the interface of SARS-CoV-2 RBD and human ACE2, many hydrophilic interactions take place, including salt bridges and hydrogen bonds.^20,52^ These closed (“down”) and open (“up”) states and the structural changes between these two conformations have also been observed on the full-length or the ectodomain of the wild-type S glycoprotein trimer.^61–63^ On the surface of SARS-CoV-2 virion, both the prefusion and postfusion S proteins are present, and their ratio varies.^61^ Therefore, the flipped-up RBD on the virion efficiently captures the ACE2 receptor on the surface of host cells for subsequent membrane fusion or endocytosis.

Closer examination of ACE2 has revealed its physiological functions. As a zinc metallopeptidase, ACE2 plays a significant role in regulating blood pressure and cardiac function^64^, converting vasoconstrictor angiotensin II to its metabolite angiotensin-(1-7).^65–67^ ACE2 knockout mice (*Ace2*^−/−^) show severe cardiac contractility defect, increased angiotensin II levels, and upregulation of hypoxia-induced genes in the heart.^65^ Moreover, cardiovascular diseases were common comorbidities during SARS-CoV and MERS-CoV infections,^5,68–73^ leading to the suspicion that SARS-CoV-2 infection might also have this same pathogenic propensity. Nevertheless, the possible destruction of ACE2 receptors induced by SARS-CoV-2 infection has not been conclusively proven by pathological examination of human samples. Therefore, importantly, the underlying molecular mechanism of heart disease caused by SARS-CoV-2 infection is still largely unknown, and it is very likely that the damages in the cardiovascular systems or other organs might not be directly linked to the ACE expression, but rather indirectly caused the harmful immune responses in the SARS-CoV-2-infected individuals.

The wide expression of ACE2 in various tissues contributes to the multi-tissue infection by SARS-CoV-2 in human. Besides its expression in lung and vasculature^74,75^, ACE2 is widely expressed in other human organs, including heart, kidney, testes, gastrointestinal tract, and brain.^74,76,77^ All of these tissues and cell types expressing ACE2 might be potential targets for SARS-CoV-2 infection. Considering the many complications associated with SARS-CoV-2 infection, significant research efforts have concentrated on elucidating SARS-CoV-2 infection in various tissues. Neurological symptoms, such as smell or taste loss, have been observed in a large majority of individuals with COVID-19.^5,78^ Recently, SARS-CoV-2 viral RNAs and proteins were demonstrated to be present in anatomically distinct regions of the brain, cerebrospinal fluid and nasopharynx,^79–81^ presenting evidence of SARS-CoV-2 neuroinvasion and neurotropism. SARS-CoV-2 nucleic acids and viral particles were also revealed in the small bowel, even in convalescent individuals,^31,82–85^ consistent with viral RNA detection in stool samples, even after negative pharyngeal swab results.^86–88^ These findings suggest that the persistence of antigen, even after functional recovery and clinical symptom resolution, is associated with the presence of virus in small intestinal epithelium. Moreover, the expression of ACE2 in spermatocytes, spermatids, and Sertoli cells enables SARS-CoV-2 infection in patients’ testes,^89–91^ leading to many uncertainties about the safety of male gametes and the risk of sexual virus transmission after SARS-CoV-2 infection.^92^ Taken together, the wide expression of ACE2 and high affinity between ACE2 and SARS-CoV-2 RBD provide high potential for SARS-CoV-2 viral infection in various tissues, and thorough studies with reliable data are therefore required.

### Other host receptors for SARS-CoV-2 attachment

**Fig. 2:**
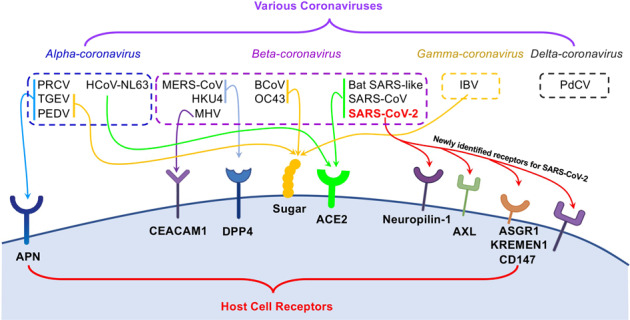
Different coronaviruses use a variety of receptors for viral attachment and entry. In the upper panel, various coronaviruses from four major genera, *alpha*-, *beta*-, *gamma*-, and *delta*-coronavirus, are in the dashed line boxes. In the lower panel, distinct receptors on the surface of host cell mediates the viral entry of the specific coronavirus. Coronaviruses: PRCV porcine respiratory coronavirus, TGEV porcine transmissible gastroenteritis coronavirus, PEDV porcine epidemic diarrhea coronavirus, MERS-CoV Middle East respiratory syndrome coronavirus, MHV mouse hepatitis coronavirus, BCoV bovine coronavirus, IBV avian infectious bronchitis coronavirus, PdCV porcine delta-coronavirus. Host cell receptors: APN aminopeptidase N, CEACAM1 carcinoembryonic antigen-related cell adhesion molecule 1, DPP4 dipeptidyl peptidase 4, ACE2 angiotensin-converting enzyme 2, ASGPR asialoglycoprotein receptor

The diversity of receptor usage is an extraordinary feature of coronaviruses (Fig. 2). Coronaviruses, as a big *Coronaviridae* family containing four classified genera, *Alpha-coronavirus*, *Beta-coronavirus*, *Gamma-coronavirus*, and *Delta-coronavirus*,^93,94^ exhibit a complex pattern for receptor recognition.^21^ Among alpha-coronaviruses, porcine transmissible gastroenteritis coronavirus (TGEV), porcine epidemic diarrhea coronavirus (PEDV), and porcine respiratory coronavirus (PRCV) recognize a receptor called aminopeptidase N (APN), a zinc peptidase.^95–98^ Among the beta coronaviruses, Middle East respiratory syndrome coronavirus (MERS-CoV) and bat coronavirus HKU4 recognize a receptor protein called dipeptidyl peptidase 4 (DPP4), a serine peptidase,^56,99,100^ while mouse hepatitis coronavirus (MHV) recognizes cell adhesion molecule CEACAM1.^101,102^ Interestingly, sugar molecules are also used as receptors or co-receptors by alpha-coronaviruses (TGEV/PEDV),^96^ beta-coronaviruses (bovine coronavirus BCoV and human coronavirus OC43),^103^ and gamma-coronavirus (avian infectious bronchitis coronavirus, IBV).^104,105^ ACE2, as a host receptor for coronavirus, is not only recognized by beta-coronavirus SARS-CoV-2,^2,48^ but also by alpha-coronavirus, human coronavirus NL63 (HCoV-NL63)^106^ and beta-coronaviruses, SARS-CoV^25,107^ and bat SARS-like coronavirus.^108^

This diversity of receptor usage by coronaviruses has inspired researchers to investigate other potential receptors for SARS-CoV-2 infection. It has been shown that SARS-CoV-2 virus cannot use APN or DPP4 as its receptor.^2^ Still, recent new studies have demonstrated that SARS-CoV-2 can use a receptor protein called neuropilin-1.^109,110^ Distinct from ACE2, which binds SARS-CoV-2 RBD directly, neuropilin-1 interacts with RRAR residues (amino acids 682–685) only after the C-terminus of SARS-CoV-2 S1 protein is exposed by protease cleavage. Therefore, neuropilin-1 serves as a “post-proteolysis receptor” for viral attachment on the surface of host cells. The RRAR peptide located in the C-terminus of SARS-CoV-2 S1 conforms to a [R/K]XX[R/K] motif, termed as “C-end rule” (CendR) peptide. It mediates viral entry by binding to neuropilin-1.^111,112^ SARS-CoV-2 can infect HEK-293T cells with neuropilin-1 alone, but with much lower infection rate compared with infectivity in HEK-293T cells stably expressing ACE2.^109^ Neuropilin-1 is a transmembrane protein with two CUB domains, two coagulation factor domains, and one MAM domain in the extracellular region.^112^ One of the coagulation factor domains containing the CendR peptide-binding site interacts directly with SARS-CoV-2 S1 CendR peptide with binding affinity of 20.3 μM at pH 7.5 and 13.0 μM at pH 5.5.^110,113^ This binding affinity is much lower than the nanomolar-level binding affinity between ACE2 and SARS-CoV-2 RBD,^47^ consistent with the observation that the neuropilin-1-dependent infection rate in vitro is significantly lower than that of ACE2.^109^ However, transcriptome analysis and immunostaining of human autopsy tissue showed abundant expression of neuropilin-1 in pulmonary and olfactory cells, but barely detectable ACE2 expression, while SARS-CoV-2 infection is positive.^18,109,114^

Tyrosine-protein kinase receptor UFO (AXL) is another candidate receptor for SARS-CoV-2 infection.^115^ AXL was identified in H1299 cells with low ACE2 expression by tandem affinity purification mass spectrometry. Overexpression of AXL promotes SARS-CoV-2 entry, while viral entry is significantly reduced in AXL-knockout cells.^115^ Among the unique characteristics of AXL and SARS-CoV-2 S protein interaction is that S protein N-terminal domain (NTD), but not RBD, mediates the interaction with AXL (882 nM binding affinity). Since AXL is not co-expressed with ACE2 in human lungs or trachea, it appears that AXL acts as an ACE2-independent receptor for SARS-CoV-2 infection.^115^ To establish the animal infection model and solve the molecular structure of NTD-AXL interaction would further promote our understanding of viral entry and provide clues for therapeutic interventions.

Using a receptor-overexpression and ligand-labeling system,^116,117^ more than 5000 human membrane proteins were screened, leading to the identification of ASGR1 and KREMEN1 as two more potential candidate receptors for SARS-CoV-2.^118^ ASGR1 interacts with both NTD and RBD, while KREMEN1 interacts with NTD, RBD, and S2 domains of the SARS-CoV-2 S protein. Their binding affinity with S protein is also in the nanomolar level at 94.8 nM and 19.3 nM for ASGR1 and KREMEN1, respectively.^118^

Several more receptor proteins, including cellular heparan sulfate, CD147, and several C-type lectin receptors, including DCL-SIGN, L-SIGN, MR, and MGL, have been evaluated for their binding with SARS-CoV-2 S protein and their contribution during SARS-CoV-2 viral infection.^119–122^ With CD147 acting as an alternative receptor for SARS-CoV-2 in ACE2-deficient cell types, cellular heparan sulfate, interestingly, is reported to be required for efficient ACE2-dependent SARS-CoV-2 infection because heparin dramatically enhances the open conformation of SARS-CoV-2 RBD for ACE2 binding.^121^ On RBD, the sulfate-binding site and ACE2-binding site are adjacent to each other to facilitate heparan sulfate-dependent enhancement of ACE2 binding.^121^ This diversity of receptor usage by SARS-CoV-2 (Fig. 2) may provide another explanation for the high infectivity of SARS-CoV-2. Consequently, these receptors may also present new opportunities as therapeutic targets to inhibit SARS-CoV-2 infection.

### Antibody-mediated SARS-CoV-2 attachment

Viruses can also invade host cells through antibody-mediated internalization of virus-antibody immune complexes. This peculiar phenomenon, called antibody-dependent enhancement (ADE) effect, has been documented for various infectious viruses, including Dengue virus, Zika virus, coronaviruses, and other viruses.^123–127^ During ADE, an antibody molecule binds a viral particle through its Fab region, while the antibody Fc region interacts with the Fc receptor (FcR) on the surface of host cells, leading to the formation of a virus-antibody-FcR complex for endocytosis.^128^ Thus, FcR-bearing cells are the main target of antibody-mediated viral entry.

During SARS-CoV infection, anti-S antibodies facilitate ACE2-independent virus internalization into various circulating immune cell types, including macrophages, monocytes, and B lymphocytes.^129–131^ Whether antibody-dependent viral entry occurs during SARS-CoV-2 infection is of fundamental importance to the understanding of viral entry and induced diseases.^123,132–135^ Although several studies have claimed that no ADE effect was observed in vitro,^136–138^ recent studies show that several antibodies, such as XG016, XG005, DH1047, DH1041, and MW05, did, indeed, induce ADE, using either pseudoviruses or authentic viruses. The phenomenon has also been reported in TZM-bl cells stably expressing human Fc*γ*R receptors, or in Raji cells, a human B cell line originally derived from a Burkitt’s lymphoma patient.^139–141^ Moreover, ADE associated with viral entry could be induced by antibodies against RBD or NTD of SARS-CoV-2 S protein. However, although the enhancement effects of anti-RBD antibodies are tightly associated with their RBD epitopes, only antibodies against certain RBD epitopes exhibit obvious ADE effect in vitro.^141^ Blockade of FcγR-binding abolishes entry ADE by SARS-CoV-2, demonstrating that virus attachment and entry are mediated by the formed antibody–FcγR complex.^139–141^

Uptake of viral particles through the ADE pathway does not necessarily result in a productive viral infection,^134,142^ but it might lead to the elevated production of proinflammatory cytokines.^5,143–146^ Similar to SARS-CoV,^129^ viral replication of SARS-CoV-2 was abortive in vitro, despite its ability to enter Raji cells through the ADE pathway.^141^ Despite the reported ADE effect in vitro and ADE in cat induced by feline infectious peritonitis virus (FIPV),^147^ animal experiments and clinical data so far suggest the unlikeliness of ADE as a successful pathway of SARS-CoV-2 infection.^140^

## Interaction between SARS-CoV-2 and host proteases

**Fig. 3:**
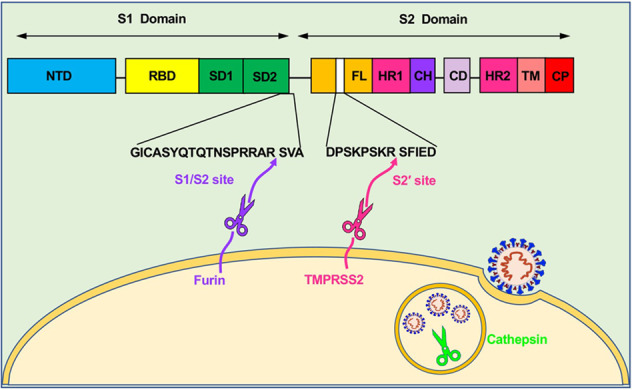
Three categories of proteases required for SARS-CoV-2 fusion and entry. Proprotein convertases (e.g., furin), cell surface proteases (e.g., TMPRSS2), and lysosomal proteases (e.g., cathepsin) participate in the proteolysis of SARS-CoV-2 S protein. Furin cleaves the multibasic site (PRRA) at the S1/S2 boundary to detach S1 from S2 domain. Subsequently, TMPRSS2 cleaves the S2’ site to further expose the internal fusion peptide for membrane fusion. Cathepsins, mainly localized in the lysosome, induce proteolysis after virion endocytosis for fusion of the viral envelope with endosomal membranes. Different domains of SARS-CoV-2 S protein (from left to right): NTD N-terminal domain, RBD receptor-binding domain, SD1 subdomain 1, SD2 subdomain 2, FL fusion loop, HR1 heptad repeat 1, CH central helix, CD connector domain, HR2 heptad repeat 2, TM transmembrane region, CP cytoplasmic tail domain

Although the hemagglutinin glycoprotein of the influenza virus undergoes proteolysis during virus packaging, the spike protein of coronaviruses is always subjected to proteolysis during viral infection, especially after binding to host cell receptors.^21,148^ Viral attachment on the cell surface initiates subsequent plasma membrane fusion or endocytosis, during which proteolysis occurs with the help of various protease activators. Importantly, the fusion peptide of coronavirus S protein is located downstream from the N-terminus of S2 stalk domain. Therefore, upon binding to host receptors, the SARS-CoV-2 S protein needs to undergo a conformational transition to expose the internal fusion peptide.^149,150^ Afterward, the exposed fusion peptide promotes either cytoplasmic or endosomal membrane fusion, leading to the ultimate accomplishment of virus entry and the release of viral RNA into the cytoplasm of host cells for translation (Fig. 3).^21,151^ Thus, proteolysis of SARS-CoV-2 S protein by various host proteases to expose the internal fusion peptide is an essential trigger for viral entry.^28,55^

### Host protease furin for SARS-CoV-2 proteolysis

Sequence analysis of SARS-CoV-2 genome revealed the presence of a 12-nucleotide or four-amino acid insertion, proline–arginine–arginine–alanine (PRRA),^152–154^ which is a consensus furin cleavage site.^155,156^ This furin cleavage site, at amino acid residues 682–685, is located at the S1/S2 boundary, and it is cleaved by furin, one of the major proteases used by SARS-CoV-2 for S protein priming (Fig. 3).^28,55^

Furin, as a type I transmembrane protein, belongs to the family of intercellular Ca^2+^-dependent serine endopeptidases, and it is also known as a proprotein convertase.^156,157^ Furin is ubiquitously expressed in various human organs and tissues, including lung, gastrointestinal tract, central nervous system, and reproductive tissues (https://www.proteinatlas.org/ENSG00000140564-FURIN/tissue).^158^ In the cytoplasmic domain for furin, targeting signals mediate the recycling of furin molecules from cell surface to the trans-Golgi network and then to the endosomal/lysosomal system.^159–161^ Meanwhile, in the extracellular region of furin, a subtilisin-like catalytic domain and a regulatory P domain are essential for its enzymatic activity.^156^ The first two substrates cleaved by furin were identified as anthrax toxin protective antigen and avian influenza virus hemagglutinin. These cleavages occurred at the surface of the host cells or in the trans-Golgi network, suggesting furin activity in distinct cellular compartments and during the activation of diverse pathogens.^162,163^ Since then, furin has been intensely investigated for its roles in protein processing of substrates during infectious diseases and even cancer progression.^157,164–166^

PRRA insertion in SARS-CoV-2 S protein harbors multiple arginine residues, thus denominated as a multibasic (or polybasic) cleavage site.^55,57^ This multibasic cleavage site has also been found in several other types of human coronaviruses, such as MERS-CoV and OC43, but not in SARS-CoV.^28^ Variants of SARS-CoV-2 have since been identified with a deleted multibasic cleavage site, indicating that PRRA insertion is not fixed during virus replication and does not affect virus replication in vitro.^167,168^ Importantly, however, when the monobasic counterpart of SARS-CoV acts as a substitute for the deleted multibasic site, furin-mediated cleavage is abrogated.^28^ Conversely, when the multibasic furin cleavage site substitutes for the monobasic site in SARS-CoV S protein, the fusion process of SARS-CoV is facilitated, even though no change in viral entry is detected in vitro.^169^ It should be noted that cleavage efficiency is not enhanced by the insertion of an additional arginine together with an alanine-to-lysine exchange.^28^ This line of evidence suggests that the multibasic motif for furin-mediated cleavage in SARS-CoV-2 S protein enables much more efficient proteolysis, making SARS-CoV-2 more aggressive than other coronaviruses, as well as providing a likely third explanation for the high infectivity and global spread of COVID-19.

### Host protease TMPRSS2 for SARS-CoV-2 proteolysis

Transmembrane serine protease 2 (TMPRSS2) is another reported protease participating in the proteolytic processing of SARS-CoV-2 S protein (Fig. 3). While furin is a type I transmembrane protein with its transmembrane domain in an N_out_–C_in_ orientation, TMPRSS2, as a type II transmembrane serine protease, adopts a N_in_–C_out_ orientation for its transmembrane domain.^170,171^ In the TMPRSS2 extracellular region, the catalytic serine protease domain is highly conserved, and several cysteines form disulfide bonds to stabilize the structure of the proteolytic domain.^149^ Transmembrane and cytoplasmic domains of TMPRSS2 contribute to its localization on the cell surface.^172^ TMPRSS2 expression is detected in basal cells of the prostate epithelium, as well as in the epithelium of a variety of other tissues, including airway epithelium, alveoli, ovaries and so on.^171,173^ However, the lack of obvious phenotype in *Tmprss2*-knockout mice has made it impossible to determine the normal physiological function of TMPRSS2, suggesting possible functional redundancy by other type II transmembrane serine proteases, or a specialized, but nonessential, contribution.^174^

Distinct from furin, which is not active for SARS-CoV, TMPRSS2 cleaves the S proteins of both SARS-CoV and SARS-CoV-2. TMPRSS2 is mainly localized on the surface of lung cells and exhibits proteolytic activity on S protein for membrane fusion.^149,175,176^ Distinct from the cleavage site at the S1/S2 boundary, the TMPRSS2 cleavage site is identified as a discrete proteolytic cleavage site within the S2 domain of S protein of both SARS-CoV and SARS-CoV-2.^177,178^ This second proteolytic cleavage site targeted by TMPRSS2, termed as the S2’ site, is located at the N-terminal S2 domain of S protein. Unexpectedly, TMPRSS2 also cleaves human ACE2 after the interaction of S protein with ACE2, inducing the shedding of ACE2 and promoting the uptake of viral particles.^179^ Mechanistically, after viral attachment, furin convertase cleaves the multibasic site at the S1/S2 boundary, removing the structural constraint of S1 on S2; subsequently, TMPRSS2 cleaves the S2’ site, leading to the release of the internal fusion peptide for the following membrane fusion.

For SARS-CoV-2 entry into host cells, proteolysis by both furin and TMPRSS2 is required for S protein activation.^180^ Upon human ACE2 receptor binding, S1 and S2 subunits of SARS-CoV-2 S protein are separated by furin-mediated cleavage at the multibasic site, and fusion peptide is further exposed by TMPRSS2-mediated cleavage at the S2’ site. Meanwhile, heptad repeat 1 (HR1, amino acids 987–1062) and heptad repeat 2 (HR2, amino acids 1263–1279) domains in S2 subunit interact with each other, leading to the formation of a six-helix bundle (6-HB) fusion core.^181–184^ The 6-HB fusion core formation brings viral particles into close proximity to the host cell membranes. Ultimately, membrane fusion occurs, and viral RNA is released into the cytoplasm.

### Host protease cathepsins for SARS-CoV-2 proteolysis

Studies on the entry mechanism demonstrated that cleavage by furin convertase is not required for SARS-CoV,^185,186^ while SARS-CoV-2 shares the same host cell receptors, but needs furin-mediated processing for viral entry.^48^ Instead, SARS-CoV virus particles enter host cells through endocytosis and are processed by cysteine proteases cathepsin B and L in lysosomes, while inhibition of cathepsin B/L blocks SARS-CoV entry.^187–189^ Moreover, host cell entry by MERS-CoV is also dependent on cathepsin L.^190^ Understanding whether (1) SARS-CoV-2 entry is also mediated by cathepsin B/L and (2) any other proteases participate in the proteolytic processing of SARS-CoV-2 S protein would help in pinpointing the molecular mechanism(s) of SARS-CoV-2 interaction with host cells and SARS-CoV-2 viral entry.

Cathepsins are a family of lysosomal proteases responsible for recycling cellular protein machineries.^191–193^ Cathepsin proteases are synthesized in the endoplasmic reticulum and translocated through Golgi apparatus to lysosomes and endosomes wherein they exhibit endopeptidase or exopeptidase activities.^194^ Because of their subcellular localization, low pH is required for their optimal enzymatic activity.

Recent studies showed the decreased viral intrusion of SARS-CoV-2 pseudoviruses after cathepsin L, but not cathepsin B, inhibition, similar to SARS-CoV and MERS-CoV.^195^ Since cathepsin L is a lysosomal protease, these results support that SARS-CoV-2 entry, like that of SARS-CoV, into host cells is mainly through endocytosis.^196^ However, in this study, only HEK293T cells overexpressing human ACE2 were used in the SARS-CoV-2 pseudovirus infection system.^195^ Infection with SARS-CoV-2 coronavirus could affect multiple organs in the human body, as explained above; therefore, the tissue tropism of SARS-CoV-2 is tightly associated with the radically different expression level of various proteases in distinct cell types and tissues. For example, in Calu-3 cells, a human lung cell line with high level of TMPRSS2 expression, but insufficient cathepsin activation, blockade of SARS-CoV-2 S cleavage at the multibasic site abolishes viral entry.^28^ On the other hand, viral entry is not affected by the same mutation blockade in Vero cells, a monkey kidney epithelial cell line with no TMPRSS2, but sufficient cathepsin protease activities.^28^ These findings are consistent with previous findings in SARS-CoV and MERS-CoV in which TMPRSS2, but not cathepsin, activity is indispensable for virus intrusion into lung cells owing to the lack of sufficient cathepsin B/L-dependent auxiliary activity in lung cells.^187,191,197,198^

In the review, we mainly discuss three proteases: furin, TMPRSS2, and cathepsins. They represent three major categories of proteases based on their active stages (Fig. 3).^21,199^ Proprotein convertases (e.g., furin) act after viral attachment of ACE2 on the surface of host cells. Cell surface proteases (e.g., TMPRSS2) act after S1 cleavage and detachment from the S2 domain of SARS-CoV-2 S protein. Lysosomal proteases (e.g., cathepsin B/L) act after viral endocytosis into the lysosome pathway of virus-targeting cells. Moreover, because of the in vivo complexity and ubiquitous infection by SARS-CoV-2, many extracellular proteases in the microenvironment and many tissue- or cell type-specific host proteases might also participate in cleaving SARS-CoV-2 S protein.

## Antiviral therapies against SARS-CoV-2 entry into host cells

**Fig. 4:**
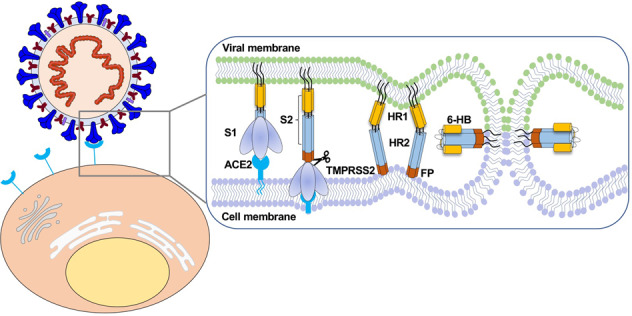
Diagram of SARS-CoV-2 entry into host cells. S protein binding to ACE2 receptor and virus attachment to the cell; S protein cleaved by TMPRSS2 produces S1 and S2 subunits. HR1 and HR2 of the S2 subunit gradually approach each other and form a six-helix bundle (6-HB), which causes the virus envelope and host cell membrane to complete fusion

Viral entry into the host cell is a multistep process (Fig. 4). First, the S protein is primed by TMPRSS2 or furin cleavage at the S1/S2 site to produce S1 and S2 subunits.^27^ The S1 subunit comprised of the RBD contributes to binding with ACE2 receptor on the target cells and stabilizes the prefusion state of the membrane-anchored S2 subunit. Then the HR1 and HR2 of the S2 subunit gradually approach each other and form a six-helix bundle (6-HB), which causes the viral envelope and host cell membrane to complete fusion.^200,201^ Upon entry to the cell, the virus is uncoated, and the RNA genome is deposited into the cytoplasm. Finally, viral genes are translated into genomic RNA and viral proteins, which are assembled together to form viral particles.^201^ Given that viral entry is a critical step for viral infection, inhibition of viral entry by targeting host- or virus‐related components was considered as the most potent strategy to prevent and treat COVID‐19.^202^ The repurposed drugs, antibodies, peptides, and small-molecule compounds for inhibiting SARS-CoV-2 entry will be systematically summarized in this section. The newest clinical status of some antibodies and small-molecule drugs was searched on COVID-19 Biologics Tracker (https://www.antibodysociety.org/covid-19-biologics-tracker/) and COVID-19 Antibody Therapeutics Tracker (https://chineseantibody.org/covid-19-track), and the results are shown in Table 1.

**Table 1:** Summary of SARS-CoV-2 antibodies and drugs in clinical trials

Name	Target	Phase	Trial ID	Sponsor	Location
*Antibodies*					
REGN-COV2 (REGN10933+ REGN10987)	RBD	3	NCT04452318	Regeneron	USA
Bamlanivimab (LY3819253, LY-CoV555)	RBD	3	NCT04497987	Eli Lilly	Canada/USA
Sotrovimab (VIR-7831/GSK4182136)	RBD	3	NCT04545060	Virbiotechnology	USA/UK
AZD7442 (AZD8895 + AZD1061)	RBD	2/3	NCT04625725 NCT04625972	AstraZeneca	UK/USA
Regdanvimab (CT-P59)	RBD	2/3	NCT04602000	Celltrion	South Korea
TY027		3	NCT04649515	Tychan	Singapore
BRII-196 + BRII-198		3	NCT04501978	Brii Biosciences	USA
DXP-593		2	NCT04551898	Beigene	China
Etesevimab (JS016, LY-CoV016, LY3832479)	RBD	2	NCT04427501	Junshi Biosciences	China/USA
*Drugs*					
Ribavirin	RdRp	1	NCT04551768	Bausch Health Americas, Inc.	NA
Remdesivir	RdRP	3	NCT04252664	Capital Medical University Chinese Academy of Medical Sciences	China
Arbidol	S	4	NCT04260594	Jieming QU	NA
Hydroxychloroquine	ACE2	4	NCT04466540	Hospital Alemão Oswaldo Cruz	Brazil
Chloroquine	ACE2	2	NCT04328493	Oxford University Clinical Research Unit, Vietnam	Vietnam
Favipiravir	RdRP	2/3	NCT04464408	King Abdullah International Medical Research Center	Saudi Arabia
Nitazoxanide	RdRP	2/3	NCT04463264	Laboratorios Roemmers S.A.I.C.F.	Argentina
Sarilumab	IL-6	2	NCT04661527	Clinica Universidad de Navarra, Universidad de Navarra	Spain
Interferon	Cytokine storm	2	NCT04465695	The University of Hong Kong	Hong Kong, China

All the sources are obtained by searching the websites of COVID-19 Biologics Tracker (https://www.antibodysociety.org/covid-19-biologics-tracker/) and COVID-19 Antibody Therapeutics Tracker (https://chineseantibody.org/covid-19-track)

### Repurposed drugs for inhibiting SARS-CoV-2 entry

Repurposing drugs from existing antiviral drugs is a fast and effective method for drug screening. Five FDA-approved drugs, including ribavirin, nafamostat, chloroquine, penciclovir, nitazoxanide, and two well-known broad-spectrum antiviral drugs, favipiravir (T-705) and remdesivir (GS5734), were identified for antiviral activity against SARS-CoV-2 by using standard methods. Among them, three nucleoside analogs, including ribavirin, penciclovir, and favipiravir through interfering with genome synthesis^203^ exhibited antiviral activity at high concentrations with EC_50_s of 109.50 μM, 95.96 μM, and 61.88 μM, respectively.^204^ Compared with other tested compounds, this is far from satisfactory. However, the antiviral activity in vivo is not always the same as that in vitro^205^. Therefore, the in vivo antiviral activity of these drugs needs to be evaluated.

Additionally, chloroquine and remdesivir showed strong antiviral activity against SARS-CoV-2 with EC_50_s of 1.13 μM and 0.77 μM in vitro, respectively.^204^ Remdesivir showed good clinical results in a clinical trial. However, side effects of remdesivir were relatively large, with adverse events reported in 32 patients (60%). The most common AEs were elevated liver enzymes, diarrhea, rash, renal impairment, and hypotension, and serious adverse events such as multiple organ dysfunction syndrome, infectious shock, and acute kidney injury were also reported.^206^ It is known that chloroquine can inhibit SARS-CoV infection by raising the endosomal pH required for virus/cell membrane fusion and interfering with the glycosylation of SARS-CoV cell receptors.^207^ Several clinical trials using chloroquine phosphate for COVID-19 have been documented in the Chinese Clinical Trials Registry.^208,209^ Although antiviral activity of chloroquine was shown in Vero cells with the SARS-CoV-2 infection, it could not block SARS-CoV-2 infection in Calu-3 cells, which express TMPRSS2.^210^ A controversial observational study on the effectiveness of chloroquine, or hydroxychloroquine, against COVID-19 has been reported.^211^ The results indicate that neither treatment has any protective effect on COVID-19 patients and that such treatment is, moreover, associated with an increased incidence of ventricular arrhythmias based on data compiled from over 600 hospitals in six continents.^211^ In view of developments, the U.S. FDA has also canceled EUA qualifications for chloroquine and hydroxychloroquine because the concluded preclinical study identified a potential drug interaction between remdesivir and chloroquine/hydroxychloroquine. Chloroquine/hydroxychloroquine may attenuate the intracellular metabolic activation process and antiviral activity of remdesivir.^212^

Nafamostat is a broad-spectrum serine protease inhibitor that targets TMPRSS2. Nafamostat showed antiviral activity against SARS-CoV-2 with an EC_50_ of 22.50 μM in vitro^204^. Also, nafamostat mesylate, a serine protease inhibitor,^213^ showed broad-spectrum neutralizing activity against SARS-CoV, MERS-CoV, and SARS-CoV-2 with EC_50_s of 1.4 nM, 5.9 nM, and 5 nM, respectively, in blocking viral infection of human lung cells.^214^ In another study, nafamostat mesylate inhibited cytopathic effect caused by SARS-CoV-2 infection in Calu-3 cells with EC_50_ of ~10 nM.^215^ A randomized clinical trial was recently launched to study the antiviral activity of nafamostat in adults with COVID-19 (ClinicalTrials.gov, NCT04352400).

Arbidol is an anti-influenza drug approved in China and Russia.^216^ It targets the SARS-CoV-2 S protein to prevent virus-mediated fusion and block the virus from entering the target cell.^217^ In cell-based assays, arbidol showed satisfactory activity against SARS-CoV-2 infection with EC_50_ of 4.11 μM in vitro.^218^ Several clinical trials for COVID-19 have been conducted to evaluate the antiviral activity of arbidol, including arbidol monotherapy and arbidol combination therapy.^219,220^ In clinical trials, post-exposure prophylaxis (PEP) using arbidol showed a certain protective effect in individuals exposed to confirmed cases of COVID-19.^220^ However, arbidol monotherapy exhibited little effect on patients hospitalized with mild and moderate COVID-19.^221^ On the other hand, the early combination of Lianhua Qingwen and arbidol may speed up the recovery of patients with moderate COVID-19 and improve prognosis.^222^

Based on high-throughput screening, six compounds (cepharanthine, abemaciclib, osimertinib, trimipramine, colforsin (NKH477), and ingenol) were repurposed from an approved drugs library by S protein-mediated pseudovirus entry assays as putative entry inhibitors. They all exhibited antiviral activity against authentic SARS-CoV-2 infection in Vero E6 cells with EC_50_s of 1.41, 3.16, 3.98, 20.52, 23.06, and 0.06 μM, respectively.^223^

Coronavirus can induce the production of interleukin (IL)-1β, IL-6, and other cytokines related to autoinflammatory diseases. Anakinra is a recombinant IL-1 receptor antagonist^224^ that may help alleviate the hyperinflammatory state associated with SARS-CoV-2, which is considered to be one of the causes of acute respiratory distress in patients. A clinical result of anakinra showed that it reduces the need for invasive mechanical ventilation in the ICU and reduces the mortality rate of severe COVID-19 patients without serious side effects.^225^ Nevertheless, confirmation of efficacy for anakinra will require controlled trials.

An analysis of a drug library containing ~12,000 clinical-stage or FDA-approved small molecules has identified a batch of candidate therapeutic drugs for COVID-19. Among them, five of the most potent compounds included apilimod (a specific PIKfyve kinase inhibitor) and four cysteine protease inhibitors: MDL-28170 (a cathepsin B inhibitor that can also weaken SARS-CoV and Ebola virus infections^226,227^), ONO 5334 (a cathepsin K inhibitor^228^), VBY-825 (a reversible cathepsin protease inhibitor^229^) and Z LVG CHN2. All were effective against SARS-CoV-2 infection in Vero E6 cells with EC_50_ values of 0.023, 0.22, 0.41, 0.3, and 0.19 μM.^230^ PIKfyve mainly exists in the early endosome and plays an important role in maintenance of endomembrane homeostasis. Therefore, apilimod has also been found to inhibit virus entry.^231^ Moreover, apilimod inhibited infection with authentic 2019-nCoV/USA-WA1/2020 virus in Vero E6 cells with an IC_50_ of ~10 nM in another report.^232^ It is worth noting that MDL-28170, ONO 5334 and apilimod inhibited virus replication in human lung cell-like cells derived from induced pluripotent stem cells (iPSC), and apilimod showed antiviral efficacy against SARS-CoV-2 infection in a primary human lung explant model.^230^

### Antibody- and protein-based antiviral therapies, convalescent plasma

#### Antibody-based antiviral therapies

##### Antibodies screened from phage library

**Fig. 5:**
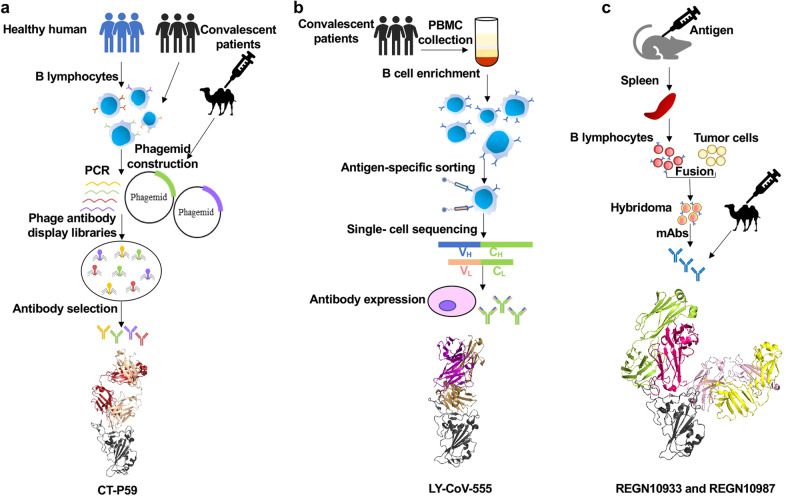
Process of preparing SARS-CoV-2 antibodies by using different technologies. **a** Phage display. **b** B cell sequencing. **c** Hybridoma. The crystal structures of the representative antibody of LY-CoV-555, CT-P59, REGN10933, and REGN10978 are shown for each technology, respectively

Single-domain antibody (sdAb) 3F11 was identified from a phage display library from nonimmune camel by using recombinant RBD of the SARS-CoV-2 S protein as antigen (Fig. 5a). Monomeric sdAb 3F11 was sufficiently potent to neutralize SARS-CoV-2 pseudovirus with an IC_50_ of 0.0038 µg/mL and for authentic SARS-CoV-2 with an IC_50_ of 0.4360 µg/mL. Competition binding assays showed that 3F11 completely blocked the binding of RBD to ACE2.^233^ The sdAb was fused with human IgG1 Fc fragments in order to overcome the limitations of monovalent sdAb.^234^ In a pseudovirus neutralizing assay, recombinant 3F11 exhibited significantly increased antiviral activity with an IC_50_ of 0.0020 µg/mL.^233^

VHH-72, which was isolated from a phage display library constructed from immunized llama with SARS-CoV S protein, has been shown to have cross-reactivity between SARS-CoV and SARS-CoV-2 S protein. Experimental data showed that the binding affinity of VHH-72 to the SARS-CoV-2 RBD was approximately 39 nM, which was weaker than that of the SARS-CoV RBD. According to this report, that result was caused by the rapid dissociation of VHH-72. Therefore, a genetic fusion of VHH-72 to the Fc domain of human IgG1 (VHH-72-Fc) was proposed for overcoming the rapid dissociation of VHH-72. VHH-72-Fc then exhibited potency against SARS-CoV-2 pseudovirus infection with an IC_50_ value of ~0.2 µg/mL.^235^

The ab1 was identified from eight large phage displayed Fab, scFv, and VH libraries by using SARS-CoV-2 RBD as antigen. In a competition assay, ab1 competes with hACE2 to bind SARS-CoV-2 RBD, indicating that it could neutralize SARS-CoV-2 through disrupting RBD-ACE2 binding. The concentration of ab1 IgG1 that completely neutralizes SARS-CoV-2 is less than 400 ng/mL in a microneutralization-based assay. And transgenic mice treated with ab1 IgG1 was protected from SARS-CoV-2 challenge.^236^ In a similar way, rRBD-15 was screened out from the phage display antibody libraries. IgG1 rRBD-15 was expressed for subsequent activity evaluation. The results showed that IgG1 rRBD-15 could bind to SARS-CoV-2 RBD with EC_50_ values of 3.8 nM in vitro and inhibit the binding of RBD to ACE2 with an IC_50_ value of 3.0 nM. In addition, IgG1rRBD-15 was sufficiently potent to neutralize SARS-CoV-2 pseudovirus infection with an IC_50_ of 12.2 nM in a pseudovirus neutralization assay.^237^

Another single-domain antibody, H11, was identified from a naive llama phage display antibody library by using SARS-CoV-2 RBD as antigen. Random mutation of H11 resulted in two mutants, H11-H4 and H11-D4, that had higher affinity to SARS-CoV-2 RBD. After bivalent Fc-nanobody fusion, H11-H4-Fc and H11-D4-Fc competed with ACE2 for RBD binding by recognizing same epitope, which partly overlaps the ACE2 binding site in RBD. Moreover, H11-H4-Fc and H11-D4-Fc could block the binding between RBD and ACE2 with IC_50_s of 61 and 161 nM, respectively. In a plaque reduction neutralization assay, they could inhibit authentic virus infection with ND_50_s (50% neutralization dose) of 6 and 18 nM, respectively.^238^

A humanized neutralizing monoclonal antibody (mAb), H014, was identified from a phage display antibody library. It could cross-neutralize SARS-CoV and SARS-CoV-2. Both IgG and Fab forms of H014 could effectively bind to SARS-CoV-2 RBD at sub-nM levels. H014 IgG exhibited antiviral activity with IC_50_ of 3 nM against SARS-CoV-2 S protein-based pseudovirus infection and with an IC_50_ of 38 nM against authentic SARS-CoV-2 in vitro. Furthermore, H014 IgG could effectively protect hACE2-Tg mice from SARS-CoV-2 infection. The epitope of H014 IgG is outside the RBM on RBD of SARS-CoV-2, indicating that H014 could block RBD-ACE2 binding through steric hindrance.^239^

In addition to camel origin sdAbs and humanized sdAbs, fully human sdAbs have also been reported.^240^ Some sdAbs were screened out by using SARS-CoV-2 S1 fragment as antigen from a fully human phage display library, followed by pseudovirus and authentic virus neutralization assay and a competition assay with ACE2 on the most potent sdAbs. Among them, n3088 and n3130, targeting a “cryptic” epitope like CR3022 in the surface of S protein, exhibited antiviral activity against SARS-CoV-2 pseudovirus infection with IC_50_s of 3.3 and 3.7 µg/mL and authentic SARS-CoV-2 infection with IC_50_s of 4.0 and 2.6 mg/mL, respectively, without cytopathic effect observed. None of them could effectively compete with ACE2.^240^

CT-P59 is an IgG form of an scFv fragment that binds to SARS-CoV-2 RBD screened from a phage library constructed from peripheral blood mononuclear cells of a convalescent patient. CT-P59 was then tested in a plaque reduction neutralization test against authentic SARS-CoV-2 and SARS-CoV-2 D614G variant. The results showed that CT-P59 effectively neutralized a SARS-CoV-2 clinical isolate in Korea with an IC_50_ of 8.4 ng/mL and reduced the replication of SARS-CoV-2 D614G variant with an IC_50_ of 5.7 ng/mL in vitro. Neutralization mechanism studies have shown that CT-P59 completely inhibits the binding of SARS-CoV-2 RBD (either wild-type or some reported mutants^241^) to ACE2. Furthermore, researchers used animal models (e.g., ferrets, hamsters, and rhesus monkeys) to evaluate the antiviral activity of CT-P59 in vivo. The results show that CT-P59 exhibits excellent virus neutralization activity in vivo. When an ADE assay was performed to investigate the possible adverse effects of CT-P59, the results showed no increase in authentic SARS-CoV-2 infection in vitro.^242^

A scFv phage display library was constructed from llama. From this phage library, nanobody NIH-CoVnb-112 was isolated, and it could block the interaction between ACE2 and SARS-CoV-2 RBD. Furthermore, NIHCoVnb-112 could effectively block the interaction between ACE2 and several high-affinity RBD variants. In the pseudovirus neutralization assay, pre-nebulization NIHCoVnb-112 exhibited potency against SARS-CoV-2 pseudovirus with an EC_50_ of 0.323 µg/mL, while the post-nebulization NIH-CoVnb-112 showed an EC_50_ of 0.116 µg/mL. Nanobodies are single-domain antibody fragments of 12–15 kD, which can be administered by inhalation and can be produced on a relatively inexpensive scale compared with other biological agents.^243^

In response to the drug demand of COVID-19, researchers have developed a rapid method for nanobody separation, including an optimized immunization protocol in alpaca combined with the surface display of E. coli in the VHH library, which allows the use of simple density gradient centrifugation of the bacterial library in a one-step selection. Finally, a single nanobody monomer, W25, was screened out. W25 bound to SARS-CoV-2 S RBD with sub-nanomolar affinity and effectively competed with receptor ACE-2 binding. In addition, W25 effectively neutralized wild-type SARS-CoV-2 and D614G variants with IC50s of 9.82 ± 1.92 nM and 5.09 ± 1.09 nM, respectively. W25FcM (W25 fused a monomeric Fc) also neutralized wild-type SARS-CoV-2 and D614G variants with IC_50_s of 27.40 ± 8.38 nM and 12.36 ± 2.84 nM, respectively. Dimeric W25Fc neutralized SARS-CoV-2 wild type and D614G variants with IC_50_s of 7.39 ± 2.39 nM and 3.69 ± 0.96 nM, respectively.^85^ In a similar method, nanobody Ty1 was screened out from an alpaca phage display library. It neutralized SARS-CoV-2 pseudotyped viruses with an IC_50_ of 0.77 µg/mL in vitro.^244^

Researchers immunized an alpaca and a llama and constructed corresponding phage display libraries, using SARS-CoV-2 RBD, or inactivated virus, as antigens to screen for single-domain antibodies with neutralizing activity against SARS-CoV-2. Among them, four nanobodies, VHH E derived from the llama and VHHs U, V, and W from the alpaca, potently neutralized infection in a dose-dependent manner. The most potent nanobody, VHH E, inhibited SARS-CoV-2 pseudovirus with an IC_50_ value of 60 nM. Plaque reduction neutralization tests were performed to identify the neutralizing activity of nanobodies with SARS-CoV-2. The four nanobodies had IC_50_ values ranging from 48 to 185 nM. X-ray crystallographic analysis showed that the four nanobodies bound to two different epitopes on RBD, namely the “E” and “UVW” interfaces, indicating that the combination of nanobodies could be synergistically targeted to inhibit infection. The neutralizing results showed VHH VE to be more effective in neutralizing than VHH E or VHH V alone. Based on the structural information, the researchers designed bivalent and trivalent nanobodies with improved neutralization properties. VHH EEE inhibited infection most effectively with IC_50_ values of 0.52 nM and 0.17 nM against SARS-CoV-2 pseudovirus and authentic virus, respectively.^245^ In addition, a large number of nanobodies have been screened as candidate drugs for the treatment of COVID-19.^246–248^

##### Antibodies screened from convalescent patients

A pool of neutralizing mAbs were screened from convalescent COVID-19 patients by using B-cell sequencing (Fig. 5b).^249^ Among them, BD-368-2 showed most effectively neutralizing activity against the pseudotyped and authentic SARS-CoV-2 infection with IC_50_s of 1.2 and 15 ng/mL, respectively.^249^ BD-368-2 also exhibited strong therapeutic and prophylactic effects in protecting transgenic mice against SARS-CoV-2 infection. Studies on the targets and mechanisms of action of BD-368-2 have shown that BD-368-2 blocks the binding of RBD to ACE2 by targeting the binding site of ACE2 in RBD.^249^

Recently, CB6 was identified from a COVID-19 convalescent patient that targets the SARS-CoV-2 RBD and blocked the binding of RBD with hACE2.^250^ The results of the neutralization assays showed that CB6 could inhibit SARS-CoV-2 pseudovirus infection with ND_50_s of 0.036, 0.023, and 0.041 μg/mL in Huh-7, Calu-3, and HEK293T cells, respectively, and effectively neutralize authentic SARS-CoV-2 infection with ND_50_ of 0.036 μg/mL in Vero E6 cells.^250^ Moreover, CB6 showed a considerable effect in both prophylactic and treatment settings of a rhesus macaque model infected with SARS-CoV-2.^250^ Mechanistic studies have showed that CB6 blocks ACE2 binding to RBD through steric hindrance and amino acid site competition.^250^

Similarly, a pair of antibodies, B38 and H4, were identified from a convalescent COVID-19 patient that exhibited antiviral activity against SARS-CoV-2 infection by using the RBD of SARS-CoV-2 as bait. The results of neutralizing assays showed that B38 had higher potency than H4 in inhibiting SARS-CoV-2 infection with IC_50_s of 0.177 and 0.896 μg/mL, respectively. In the crystal structure analysis, the target of B38 overlaps with the binding site of ACE2 in RBD, suggesting that B38 functionally mimics ACE2 to bind RBD to block RBD-ACE2 interaction. In the competition assay, both B38 and H4 competed with ACE2 to bind RBD. Furthermore, B38 and H4 showed excellent antiviral activity in vivo. While no competition between B38 and H4 was observed, suggesting that the two neutralizing mAbs act on different RBD sites. Therefore, B38 and H4 can be used as an ideal mAb pair for virus targeting to avoid immune escape in future clinical applications.^251^

A pool of neutralizing mAbs targeting SARS-CoV-2 RBD was screened from the PBMCs of SARS-CoV-2-infected individuals. Among them, P2C-1F11 and P2B-2F6 neutralized SARS-CoV-2 pseudovirus with IC_50_s of 0.03 and 0.05 µg/mL, respectively. Consistent with this result, P2C-1F11 and P2B-2F6 had IC_50_s of 0.03 and 0.41 μg/mL, respectively, in an authentic SARS-CoV-2 neutralization assay.^252^ In a similar study, neutralizing mAbs were identified from convalescent patients by using high-throughput screening with specific antigen. Among the identified mAbs, CC12.1 effectively neutralized the pseudotyped and authentic SARS-CoV-2 infection with IC_50_s of 0.019 and 0.022 µg/mL, respectively. Moreover CC12.1 showed complete protection to Syrian hamsters against SARS-CoV-2 infection. Further, mapping assay showed that CC12.1 targets SARS-CoV-2 RBD and blocks receptor ACE2 binding.^253^

Similarly, COVA1-18 and COVA2-15 were identified from convalescent patients by using SARS-CoV-2 S protein as antigen. Both neutralizing mAbs target the RBD of SARS-CoV-2 that neutralized SARS-CoV-2 pseudovirus with an IC_50_ of 8 ng/mL. Further, COVA1-18 and COVA2-15 had IC_50_ values of 7 and 9 ng/mL, respectively, in authentic SARS-CoV-2 neutralizing assay.^254^ Similarly, a neutralizing antibody, EY6A, which was isolated from a convalescent patient, targets the conserved sequence of SARS-CoV-2 RBD, but distant from the ACE2 binding site^255^. Neutralizing antibodies MW05 and MW07, which had high RBD-binding abilities and strong RBD/ACE2-disrupting activities, showed neutralizing activities with NT_50_s of 0.030 and 0.063 μg/mL, respectively, against SARS-CoV-2 pseudovirus. Furthermore, MW05 and MW07 blocked authentic SARSCoV-2 entry into Vero E6 cells with NT_100_s of ~1 μg/mL and 5 μg/mL, respectively. In order to eliminate ADE, researchers introduced the LALA mutation to the Fc region of MW05, after which MW05/LALA showed prophylactic and therapeutic efficacy against SARS-CoV-2 in rhesus monkeys.^139^

A batch of cross-reactive monoclonal neutralizing antibodies against SARS-CoV, SARS-CoV-2, and WIV1 were identified from SARS convalescent patients. ADI-55689, and ADI-56046 showed neutralization activity against SARS-CoV-2 pseudovirus with IC_50_s of 0.05–1.4 μg/mL. Similar IC_50_ values were observed in authentic SARS-CoV-2^256^. Diversity was introduced into the variable genes of the heavy chain and light chain of ADI-55688, ADI-55689 and ADI-56046 by oligonucleotide-based mutagenesis for affinity optimization and then transformed into *Saccharomyces cerevisiae* by homologous recombination to generate a yeast display library. ADG-2, one of the affinity-matured variants, was the most potent in neutralizing an alternative authentic SARS-CoV-2 with an IC_50_ of ~1 ng/mL. ADG-2 also showed complete protection against respiratory burden and viral replication in the lungs and lung pathology in an immunocompetent mouse model of COVID-19. Structural and biochemical studies exhibited that ADG-2 recognizes a highly conserved epitope overlapping SARS-CoV-2 RBD.^257^

Some neutralizing mAbs were identified from SARS-CoV-2 convalescent patients by using SARS-CoV-2 S protein.^258^ These neutralizing mAbs had antiviral activity with IC_50_s in the range of 15–4000 ng/mL in a neutralizing assay against SARS-CoV-2 strain WA1/2020. Among them, both COV2-2196 and COV2-2130 could completely block the binding between SARS-CoV-2 RBD and receptor ACE2 in a competition assay, even though epitopes were different on SARS-CoV-2 RBD. Therefore, COV2-2196 and COV2-2130 could simultaneously bind to S protein. Furthermore, COV2-2196 and COV2-2130 used in combination or alone could protect BALB/c mice and non-human primate from SARS-CoV-2 infection.^259^

A pool of neutralizing antibodies was isolated from PBMCs of five severe COVID-19 patients. Nine of them were potent enough to neutralize authentic SARS-CoV-2 with IC_50_ values in the range of 0.7–9 ng/mL. Among these antibodies 2–15, etc. target SARS-CoV-2 RBD, and 2–17, etc. target SARS-CoV-2 NTD, while 2–43 and 2–51 target a quaternary epitope on the top of SARS-CoV-2 RBD. The most potent antibody, 2–15, had IC_50_ values of 5 and 0.7 ng/mL, respectively, against pseudotyped and authentic SARS-CoV-2 infection. Further, 2–15 exhibited effective protection against SARS-CoV-2 infection in a golden Syrian hamster model.^260^

S309, which was identified from the peripheral blood of SARS-infected patients, had cross-reactivity for SARS-CoV-2. In a pseudovirus neutralization assay, S309 could effectively neutralize both pseudoviruses. And S309 could effectively neutralize both pseudoviruses and potently neutralize authentic SARS-CoV-2 (2019n-CoV/USA_WA1/2020) with an IC_50_ value of 79 ng/mL by targeting the RBD. By mapping epitopes, it was shown that S309 could recognize a highly conserved epitope in the SARS-CoV-2 RBD, comprising the N343-glycan.^261^

By investigating antibody responses in COVID-19 patients at different periods, researchers could screen out some mAbs that efficiently neutralized SARS-CoV-2. For example, C121, C144, and C135 were sufficiently potent to neutralize authentic SARS-CoV-2 with IC_50_s of 1.64, 2.55, and 2.98 ng/mL, respectively.^262^

CV30 showed neutralizing activity against SARS-CoV-2 pseudovirus with an IC_50_ value of 0.03 μg/mL.^263^ Further, crystal structure of CV30 revealed that the epitope of CV30 overlapped the receptor ACE2 binding motif in SARS-CoV-2 RBD. The neutralization experiment results showed that CV30 could effectively neutralize SARS-CoV-2 infection with an IC_50_ of 0.118 μg/mL. CV30 could also induce the shedding of the S1 subunit to reduce viral infection. A germline reversion of CV30 showed weaker binding activity and neutralizing activity against SARS-CoV-2 pseudovirus, indicating that the appropriate somatic mutation is needed for neutralization activity of antibodies against SARS-CoV-2.^264^

LY-CoV555, a potent neutralizing antibody targeting SARS-CoV-2 RBD from a convalescent COVID-19 patient, was screened out through high-throughput microfluidic screening of antigen-specific B-cells. In order to test the antiviral activity of the selected antibodies, a pseudovirus neutralization assay, a replication-capable virus neutralization assay with a reporter gene, and an authentic virus (the Italian INMI-1 isolate and the USA/Wa-1/2020 isolate) neutralization assay were all performed. LY-CoV555 exhibited strong neutralizing activity against SARS-CoV-2 in these assays, and LY-CoV555 neutralized both isolates with IC_50_s of <100 ng/mL. In addition, LY-CoV555 exhibited effective protection in prophylaxis doses as low as 2.5 mg/kg in rhesus macaques.^265^ Further, the results of a clinical trial of LY-CoV555 showed that patients treated with LY-CoV555 reduced the hospitalization of patients. In addition, the symptom burden of high-risk groups was comparable to that of placebo, and the safety was similar to that of placebo.^39^

##### Antibodies identified from immunized animal

7B11and 18F3 were identified from previously screened antibodies specific to SARS-CoV. Both of them neutralized about 80% SARS-CoV-2 pseudovirus infection at 10 μg/mL. And 7B11 blocked the binding of SARS-CoV-2 RBD to ACE2 owing to the proximity between epitope and ACE2 binding site. However, 18F3 could not block the binding of SARS-CoV-2 RBD to ACE2 owing to the distance between epitope and ACE2 binding site.^266^

A large number of fully human mAbs targeting different sites in SARS-CoV-2 RBD were isolated from the plasma of immunized transgenic mice and COVID-19 convalescent patients (Fig. 5c). Among them, REGN10933 and REGN10987 could effectively block the binding of ACE2 to the RBD and neutralize SARS-CoV-2 at pM level. The crystal structure showed that these two antibodies target different sites of SARS-CoV-2 RBD. Therefore, these two antibodies can be used in combination as a cocktail.^267^ In addition, viral escape mutations could be effectively avoided in such antibody cocktail (REGN-CoV2) therapy.^268^ Researchers have also evaluated the antiviral activity of REGN-CoV2 in rhesus macaques and hamsters. When administered prophylactically or therapeutically in rhesus monkeys, results showed that REGN-COV-2 could make a substantial reduction in the viral load of the upper and lower respiratory tracts of rhesus monkeys and reduce the pathological sequelae caused by the virus. Similarly, hamster administration could limit weight loss, as well as reduce pneumovirus titers and pneumonia.^269^ In an interim analysis of a clinical trial, REGN-COV2 reduced the viral load of patients and had a greater impact on patients who had not yet started an immune response or had a high baseline viral load. The safety results of the combined REGN-COV2 dose group and the placebo group were similar.^40^

Similarly, many chimeric neutralizing mAbs that having cross-reactivity against SARS-CoV-2 and SARS-CoV were identified from transgenic mice immunized with SARS-CoV S protein. Among them, 47D11 was reconstructed and expressed in human IgG1. Further study, humanized 47D11 showed binding activity with SARS-CoV-2 S protein that were expressed on the cell surface. And humanized 47D11 neutralized the pseudotyped and authentic SARS-CoV-2 infection with IC_50_s of 0.061 and 0.57 μg/mL, respectively. The 47D11 also targets the RBD verified by an ELISA.^270^

Using SARS-CoV-2 RBD fused with a mouse IgG Fc as the antigen, a batch of mAbs specifically targeting SARS-CoV-2 RBD was obtained using animal immunization and hybridoma technology. RBD binding and SARS-CoV-2 pseudovirus neutralization assays were performed on these antibodies. Researchers humanized the antibodies with strong neutralizing activity, and a humanized version of the 2H2/3C1 cocktail was found to potently neutralize authentic SARS-CoV-2 infection in vitro with an IC_50_ of 12 ng/mL with a protective effect on mice in 24 h post-infection. The crystal structure showed that 2H2 and 3C1 target two different epitopes in SARS-CoV-2 RBD and only weakly compete for binding to RBD.^271^ A recent study tested the antiviral activity of multiple antibodies from different research teams against infection by SARS-CoV-2 variants B.1.1.7 emerged in the UK and B.1.351 emerged in South Africa. The results showed that mAb cocktails maintained better neutralizing activity against these mutant strains than using a single antibody.^272^

#### Convalescent plasma

In convalescent plasma therapy, plasma is collected from recovered patients and transfused to symptomatic patients. The transfer of convalescent plasma is an ancient concept that has been used since the time of the Spanish flu pandemic in 1918.^273^ During the SARS pandemic in 2003, convalescent plasma was successfully used,^274,275^ as well as during the influenza H1N1 pandemic in 2009^276^ and the Ebola outbreak in Africa in 2015.^277^ Several small observational studies published during the COVID-19 pandemic indicate that convalescent plasma is part of an effective treatment strategy for severe disease.^278–282^ Meanwhile, a study suggested that administration of convalescent plasma late in the disease course was ineffective in reducing mortality.^283^ A recent report showed that the virus strain with ΔH69/ΔV70 and D796H appeared in the course of plasma treatment and that this mutant strain was not only less sensitive to some existing antibodies, but also enhanced infection.^284^ The clinical effect of plasma therapy is still unclear; therefore, clinical application should be strictly monitored. There is no accepted best method for measuring antibodies in plasma, and plasma antibody titers vary widely among COVID-19 recovered patients; in addition, hospitalized COVID-19 patients may already have SARS-CoV-2 neutralizing antibody titers comparable to plasma donors, which would limit the benefit of plasma from recovered patients in this patient population.

### Peptide-based antiviral therapies

#### Peptides targeting RBD

**Fig. 6:**
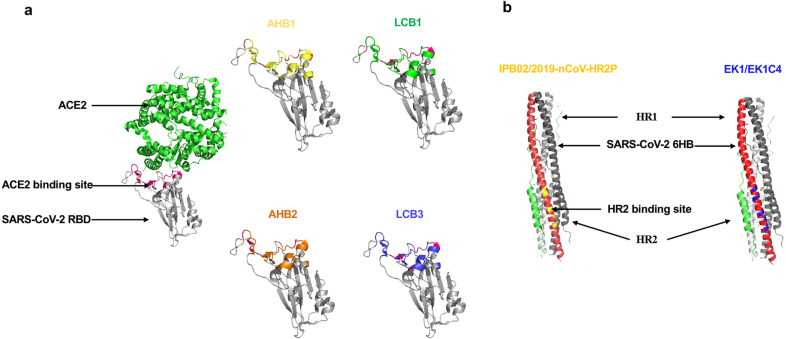
Peptides targeting the SARS-CoV-2 S protein. **a** RBD-targeting peptides: targets of AHB1, AHB2, LCB1, and LCB3 were shown in yellow, orange, green, and blue, respectively, in SARS-CoV-2 RBD. **b** 6-HB-targeting peptides: targets of IPB02 and 2019-nCoV-HR2P overlaps with the HR2 binding site that colored in yellow. The targets of the pan-CoV fusion inhibitors, EK1 and EK1C4 peptides, was shown in blue

The binding of SARS-CoV-2 RBD with ACE2 is an inevitable event that induces virus invasion. Consequently, disrupting the contact and binding between RBD and ACE2 is a key strategy for antiviral therapy. The efficient binding of RBD and ACE2 indicates that peptides derived from the ACE2 may have the same, or even more, binding activity to RBD. Disrupting SARS-CoV-2-RBD binding to ACE2 with peptides has the potential to inhibit the virus from entering human cells, presenting a new modality for therapeutic intervention (Fig. 6). Detailed information on peptide-based antivirals was displayed in Table 2.

**Table 2:** Summary of peptides showing SARS-CoV-2 entry inhibition

Name	Description	Sequence	Target	Inhibitory activity (IC_50_)	Clinical status	Ref
SBP1	A peptide derived from ACE-PD α1 helix	IEEQAKTFLDKFNHEAEDLFYQS	RBD	–	Preclinical	^280^
AHB1	Derived from ACE2 Deep sequencing identified three ACE2 helix scaffolded designs	DEDLEELERLYRKAEEVAKEAKDASRRGDDERAKEQMERAMRLFDQVFELAQELQEKQTDGNRQKATHLDKAVKEAADELYQRVRELEEQVMHVLDQVSELAHELLHKLTGEELERAAYFNWWATEMMLELIKSDDEREIREIEEEARRILEHLEELARK	RBD	LV: IC_50_ = 35 nM	Preclinical	^281^
AHB2	A truncate of AHB2	ELEEQVMHVLDQVSELAHELLHKLTGEELERAAYFNWWATEMMLELIKSDDEREIREIEEEARRILEHLEELARK	RBD	LV: IC_50_ = 15.5 nM	Preclinical	^281^
LCB1	De novo interface designs	DKEWILQKIYEIMRLLDELGHAEASMRVSDLIYEFMKKGDERLLEEAERLLEEVER	RBD	LV: IC_50_ = 23.54 pM	Preclinical	^281^
LCB3	De novo interface designs	NDDELHMLMTDLVYEALHFAKDEEIKKRVFQLFELADKAYKNNDRQKLEKVVEELKELLERLLS	RBD	LV: IC_50_ = 48.1 pM	Preclinical	^281^
IPB02	A lipopeptide made by adding a cholesterol group to the C-terminal of IBP01	ISGINASVVNIQKEIDRLNEVAKNLNESLIDLQELK (Chol)	S2-HR1	PsV: IC_50_ = 0.08 μM	Preclinical	^293^
EK1	Derived from HR2 (OC43)	SLDQINVTFLDLEYEMKKLEEAIKKLEESYIDLKEL	S2-HR1	PsV: IC_50_ = 2.375 μM	Preclinical	^289^
				LV: IC_50_ = 2.468 nM		
EK1C4	cholesterol group to the C-terminal of EK1	SLDQINVTFLDLEYEMKKLEEAIKKLEESYIDLKELGSGSG-PEG4 (Chol)	S2-HR1	PsV: IC_50_ = 15.8 nM	Preclinical	^289^
				LV: IC_50_ = 36.5 nM		
2019-nCoV-HR2P	HR2 (1150–1185)	DISGINASVVNIQKEIDRLNEVAKNLNESLIDLQEL (aa1168–1203)	S2-HR1	PsV: IC_50_ = 0.98 μM	Preclinical	^200^

*PsV* pseudovirus, LV live virus

SBP1, a 23-mer peptide fragment derived from human ACE2 peptidase domain (PD) α1 helix composed entirely of ACE2 amino acids, was chemically synthesized. Bio-layer interferometry was performed to investigate the binding activity between SARS-CoV-2 RBD and SBP1. The result showed that SBP1 specifically bound with the SARS-CoV-2 RBD at K_D_ = 47 nM.^285^ Therefore, SBP1 may have the potential to inhibit virus entry into cells.

Peptides AHB1, AHB2, LCB1, and LCB3 were screened out using two methods, one design based on ACE2 binding to RBD and another de novo design based on the RBD binding surface. First, researchers designed a pool of peptides for the two methods. Then these peptides were screened for binding to RBD displayed on the surface of yeast cells. Three and 105 peptides derived from method 1 and 2, respectively, with high binding activity were further sequenced. The active peptides were subjected to PCR mutation, and mutants AHB1, AHB2, LCB1, and LCB3 were obtained. Prokaryotic expression of these peptides was performed, and the RBD binding activity of the expressed products was detected. The results showed that LCB1 and LCB3 showed binding signals at 5 pM of RBD. For peptide thermostability testing, MT values for most were greater than 90 °C. After 14 days at room temperature, these peptides retained full binding activity. In the crystal structure of peptide combined with SARS-CoV-2 RBD, LCB1 and LCB3 formed multiple hydrogen bonds and salt bridges with the RBD with buried RBD surface areas.^286^ The crystal structure and peptide sequence are shown in the figure.^287^ Finally, the neutralizing activity of AHB1, AHB2, and LCB1 to LCB5 against SARS-CoV-2 was tested. AHB1 and AHB2 strongly neutralized SARS-CoV-2 with IC_50_s of 35 and 15.5 nM, respectively, while LCB1 and LCB3 were much more potent, with IC_50_s of 23.54 and 48.1 pM, respectively. The entire span of time for peptide development took about 4 months, noting that rapid drug development is essential during the spread of severe epidemics. Therefore, the two methods could be considered candidates for quick drug development.^286^

#### Peptides Targeting HR1

Peptides derived from the CHR or NHR of HIV have been revealed to possess antiviral activity through blocking the formation of 6-HB^288^. Such peptides have also appeared in coronavirus research, including SARS-CoV, and MERS-CoV.^289–292^ EK1, a pan-CoV fusion inhibitor derived from the HR2 of HCoV-OC43, exhibited inhibitory activity against diverse HCoVs, including HCoV-229E, HCoV-NL63, HCoV-OC43 SARS-CoV and MERS-CoV.^293^ With the outbreak of COVID-19, EK1 was identified to effectively inhibit SARS-CoV-2 S protein-mediated membrane fusion with an IC_50_ of 0.19 µM and pseudovirus infection with an IC_50_ of 2.38 µM in a dose-dependent manner.^200,294^ At the same time, researchers synthesized 2019-nCoV-HR2P (aa1168–1203), and it could effectively inhibit SARS-CoV-2 membrane fusion with an IC_50_ of 0.19 µM and pseudovirus infection with an IC_50_ of 0.98 µM.^200^ Emerging studies showed that lipid conjugation is a viable strategy to enhance antiviral activity and stability of peptide-based fusion inhibitors.^295–297^ Researchers further modified EK1 and obtained EK1C4, which effectively inhibited membrane fusion and pseudovirus infection with IC_50_s of 1.3 and 15.8 nM^294^. Similarly, IPB02 was designed as a SARS-CoV-2 fusion inhibitor, and it showed highly potent activity in inhibiting SARS-CoV-2 S protein-mediated cell-cell fusion and pseudovirus infection with IC_50_s of 0.025 µM and 0.08 µM.^298^

### Small-molecule compound-based antiviral therapies

#### Small molecules targeting S protein

The S protein of SARS-CoV-2 plays a key role in receptor recognition and virus-cell membrane fusion. Therefore, S protein is also a key target for antiviral therapy. A hydrophilic compound, Salvianolic acid C (Sal-C) isolated from Danshen, a traditional Chinese medicine (TCM), inhibited SARS-CoV-2 infection by blocking the formation of 6-HB core with an EC_50_ of 3.41 μM in authentic SARS-CoV-2 inhibition assays^299^. In addition, two novel drug-like compounds, DRI-C23041 and DRI-C91005, showed antiviral activity in disrupting the interaction between hACE2 and SARS-CoV-2 S protein. DRI-C23041 also inhibited the entry of SARS-CoV-2-S pseudovirus into ACE2-expressing cells with an IC_50_ of 5.6 μM^300^.

#### TMPRSS2 inhibitors

The virus attaches to the receptor ACE2 through the S protein, and the host cell serine protease TMPRSS2 triggers cleavage and conformational changes of S protein, thereby triggering viral invasion. Therefore, TMPRSS2 could be used as a target to prevent virus entry. Camostat mesylate, a clinically proven TMPRSS2 inhibitor, significantly inhibited SARS-CoV-2 pseudovirus entry into Calu-3 cells with an EC_50_ of ~1 μM and CC_50_ > 500 μM^301^. Similarly, camostat mesylate significantly reduced authentic SARS-CoV-2 infection in Calu-3 cells and reduced SARS-CoV-2 pseudovirus infection in primary human lung cells^301^. A randomized clinical trial is under evaluation for camostat mesylate as a treatment for SARS-CoV-2 (Phase IIa, ClinicalTrials.gov, NCT04321096). Double-blinded, randomized, placebo-controlled trials are being carried out on 334 COVID-19 patients (phase IV, ClinicalTrials.gov, NCT04338906). In addition, bromhexine, a generic mucolytic targeting TMPRSS2^302^, is currently being investigated clinically for SARS-CoV-2 (ClinicalTrials.gov, NCT04273763, NCT04340349). MI-432 and MI-1900 are two prospective peptide mimetic inhibitors of TMPRSS2^303^. Both exhibited antiviral activity against SARS-CoV-2 infection in vitro^303^.

#### Cathepsin B/L inhibitors

In addition to TMPRSS2, cellular cathepsins can also prime viral S protein cleavage and favor viral fusion. Of interest are cathepsin B and cathepsin L, which become active in the early and late endosome, respectively, and are known activators for fusion.^304,305^ P9, derived from mouse β-defensin-4, has broad-spectrum antiviral activity against multiple respiratory viruses by interfering with cathepsin L.^306^ The P9-optimized product P9R showed antiviral activity against SARS-CoV-2 with an IC_50_ of 0.9 μg/ml in a plaque reduction assay.^307^ Further, an eight-branched derivative, 8P9R, showed more potent antiviral activity with an IC_50_ of 0.3 μg/ml. The 8P9R can inhibit both endocytic and surface pathways of SARS-CoV-2 mediated by TMPRSS2 by aggregating virus particles. In vivo, 8P9R alone, or in combination with other drugs (arbidol, chloroquine, and camostat), could significantly inhibit SARS-CoV-2 replication in hamsters.^308^ E64-d, a broad cathepsin B/L inhibitor, showed inhibitory activity with an IC_50_ of ~4.487 μM in a SARS-CoV-2 pseudovirus infection assay.^301,309^ Teicoplanin, an antibiotic currently used for the treatment of Gram-positive bacterial infections, had antiviral activity against SARS-CoV, MERS-CoV, and Ebola virus in vitro.^310,311^ Teicoplanin acts on the early step of the coronavirus viral life cycle by directly inhibiting the enzymatic activity of cathepsin L. Teicoplanin inhibited SARS-CoV-2 pseudovirus infection with an IC_50_ of 1.66 µM, which is much lower than the commonly used dose of 8.78 µM used to inhibit Gram-positive bacteria.^312^ More investigation of teicoplanin was encouraged for the treatment of COVID-19 disease.^310^

## Conclusions and prospects

In this article, we reviewed the molecular mechanisms of interaction between SARS-CoV-2 virus and host cells, especially receptor-mediated virus attachment on the surface of host cells and protease-mediated proteolysis during virus entry. Obviously, many steps are involved in successful viral infection, including viral attachment on receptors on the cell surface, proteolysis or subsequent lysosomal proteolysis after endocytosis, 6-HB formation after the exposure of the internal fusion peptide, and membrane fusion, followed by the release of viral RNA to the cytoplasm of host cells. Although the complexity of viral entry might at first glance appear to be inefficient, SARS-CoV-2 exhibits extraordinarily high transmissibility. Recent studies using structural analysis, as well as molecular and cellular techniques, explain the reasons for the high infectivity of SARS-CoV-2 by revealing the exceptional binding affinity between SARS-CoV-2 RBD and its receptor ACE2, the diversity of receptor usage by SARS-CoV-2, and the multibasic motif at the S1/S2 boundary of SARS-CoV-2 S protein for efficient proteolysis.

In this review, we also highlighted the interventional therapies targeting the SARS-CoV-2 viral entry machineries, including its receptors and proteases. Numerous treatments, including small-molecule compounds, antibodies, and antiviral peptides, are proposed and under intensive investigations. Studies to unravel the molecular mechanism of interactions between SARS-CoV-2 virus and host cells provide profound insights into prevention and treatment approaches. However, the complexity of viral entry and the functional redundancy of receptors and proteases may suggest the unlikeliness of using just one drug to fully inhibit infection, especially considering the ability of coronaviruses to manifest in different tissues. For example, a complete blocking of ACE2 receptor could not inhibit infection in certain cell types owing to the reported ACE2-independent receptor.^115^ Moreover, some extracellular proteases or cell type-specific lysosomal proteases could compensate for the lack of furin or TMPRSS2.^21^ Therefore, effective treatment options will need extensive clinical trials and post-approval monitoring.

Recently, various SARS-CoV-2 variants have been reported, including the emerging new SARS-CoV-2 lineages B.1.1.7 in England and B.1.351 in South Africa.^313^ At this time, researchers are using all resources to test the efficacy of various vaccines and drugs against these new COVID variants with diverse escape mutations.^314^ How these mutations induce more transmissivity, whether they induce higher morbidity and mortality, and how they systematically affect interactions between SARS-CoV-2 and host cells are largely unknown. Therefore, the development of broad-spectrum antiviral drugs and vaccines against SARS-CoV-2 and its variants will require a long-term strategy in the search for clinical treatments.
